# Pneumococcal infection of respiratory cells exposed to welding fumes; Role of oxidative stress and HIF-1 alpha

**DOI:** 10.1371/journal.pone.0173569

**Published:** 2017-03-09

**Authors:** Jonathan Grigg, Lisa Miyashita, Reetika Suri

**Affiliations:** Centre for Genomics and Child Health, Blizard Institute for Cell and Molecular Sciences, London, United Kingdom; University of Alabama at Birmingham, UNITED STATES

## Abstract

Welders are more susceptible to pneumococcal pneumonia. The mechanisms are yet unclear. Pneumococci co-opt the platelet activating factor receptor (PAFR) to infect respiratory epithelial cells. We previously reported that exposure of respiratory cells to welding fumes (WF), upregulates PAFR–dependent pneumococcal infection. The signaling pathway for this response is unknown, however, in intestinal cells, hypoxia-inducible factor-1 α (HIF 1α) is reported to mediate PAFR-dependent infection. We sought to assess whether oxidative stress plays a role in susceptibility to pneumococcal infection via the platelet activating factor receptor. We also sought to evaluate the suitability of nasal epithelial PAFR expression in welders as a biomarker of susceptibility to infection. Finally, we investigated the generalisability of the effect of welding fumes on pneumococcal infection and growth using a variety of different welding fume samples. Nasal epithelial PAFR expression in welders and controls was analysed by flow cytometry. WF were collected using standard methodology. The effect of WF on respiratory cell reactive oxygen species production, HIF-1α expression, and pneumococcal infection was determined using flow cytometry, HIF-1α knockdown and overexpression, and pneumococcal infection assays. We found that nasal PAFR expression is significantly increased in welders compared with controls and that WF significantly increased reactive oxygen species production, HIF-1α and PAFR expression, and pneumococcal infection of respiratory cells. In unstimulated cells, HIF-1α knockdown decreased PAFR expression and HIF-1α overexpression increased PAFR expression. However, in knockdown cells pneumococcal infection was paradoxically increased and in overexpressing cells infection was unaffected. Nasal epithelial PAFR expression may be used as a biomarker of susceptibility to pneumococcal infection in order to target individuals, particularly those at high risk such as welders, for the pneumococcal vaccine. Expression of HIF-1α in unexposed respiratory cells inhibits basal pneumococcal infection via PAFR-independent mechanisms.

## Introduction

Epidemiological studies suggest that welders are at increased risk of respiratory infections in general and specifically pneumococcal pneumonia[1]. Originally reported in national analyses of occupational mortality, increased risk of pneumococcal pneumonia in welders was subsequently found in a large case–controlled study of men admitted to hospitals in the English West Midlands with community-acquired pneumonia, and recently confirmed in epidemiological studies in both the UK and other countries[2–5],[6–8]. Increased pneumonia risk is associated with recent exposure, and is not found in former welders after normal retirement age[3–5].

In a previous study we found that *in vitro* exposure of respiratory cells to mild-steel welding fumes (MS-WF) upregulates the expression of the platelet activating factor receptor (PAFR) which is, in turn, co-opted by pneumococci to infect human respiratory cells[9],[10]. In this interaction, phosphatidyl choline in the pneumococcal cell wall acts as a molecular mimic of human PAF, and the bacterium utilises this to both adhere to the cell surface, then infect cells as the receptor is internalised[9]. Compatible with previous reports for other stimuli[11,12], we found that PAFR-dependent infection determines only welding fume (WF)-stimulated adhesion, and not “basal” infection of unstimulated cells[10]. We also identified a role for WF-induced oxidative stress since pneumococcal infection to respiratory cells was blocked by the anti-oxidant N acetyl cysteine (NAC)[10]. However, the intracellular signaling pathway for WF-induced pneumococcal infection, and PAFR expression in active welders remains unclear.

A putative role of the hypoxia inducible factor 1 alpha (HIF-1α, the major transcriptional regulator of cellular responses to hypoxia[13]), in mediating upregulation of PAFR expression is suggested by Keely et al[14] who reported that in gut epithelial cells, PAFR mRNA and protein expression is rapidly induced by hypoxia, blocking PAFR significantly reduced *E*. *Faecalis* translocation across the epithelial barrier, that HIF-1α has a major role in the induction of PAFR and *E*. *Faecalis* translocation, and that there is a binding site for HIF-1α in the proximal PAFR promoter.

In this study, we hypothesised that increased pneumococcal infection in respiratory cells exposed to WF, is via oxidative stress induced HIF-1 α, which in turn upregulates PAFR-dependent adhesion and infection. We therefore sought to assess i) whether constitutive PAFR expression is increased in nasal epithelial cells from welders, ii) whether the effect of WF on pneumococcal infection is generalisable to WF with differing compositions, and iii) the role of oxidative stress induced HIF-1α in this response.

## Materials and methods

### Welding fumes

WF were a gift from The Welding Institute (Cambridge, UK). These WF were obtained using a standardised method in accordance with the International Standard (ISO) 15011–1:2009, as previously described [12]. Briefly, manual metal arc welding electrodes E7018 basic type were run to produce a weld bead inside a fume collection system. Welding fumes with a mode particle diameter 6.8 μm [13], were extracted through the hood on top of the box, collected on a filter paper, removed by brushing, and stored in airtight glass containers. The composition of the WF was assessed after digestion in nitric/hydrochloric acid in a high temperature closed vessel microwave assisted dissolution system. Analysis was done using inductively coupled plasma–atomic emission spectroscopy.

Metal compositions of WF (weight %) are described in Table 1. The mild steel WF sample had a standard composition for the type WF i.e % of chromium is less than 18%[15]. Chromium, iron, manganese and nickel are generally the major metal components of WF. Thus samples in which each metal was either the most abundant or at least doubly abundant than other samples available, were analysed to study the generalisability of the effect of welding fumes on pneumococcal infection and growth. WF samples were suspended in phosphate buffered saline (PBS) for use in experiments. A standard dose of 200μg/ml was used for 2h which is similar to that estimated by Phalen et al (85 mg/cm^2^) for hot spots of inhaled PM deposition on respiratory cells[16] and does not have an effect on cell viability[10].

**Table 1:** Metal compositions of the WF samples used in the study by % weight per sample. MS-WF is our standard WF sample.

Weight (% of sample)	Mild Steel	Chromium-rich	Iron-rich	Manganese-rich	Nickel-rich
Aluminium	0.330551	0.4	0.2	0.3	5.9
Barium	<0.1	<0.1	<0.1	<0.1	<0.1
Bismuth	<0.1	<0.1	<0.1	<0.1	<0.1
Calcium	8.07181	1	0.5	1.3	3.2
Cobalt	<0.1	<0.1	<0.1	<0.1	<0.1
Chromium	<0.1	11.7	4.2	4.1	<0.1
Copper	<0.1	<0.1	<0.1	<0.1	<0.1
Iron	20.83141	36.7	54.2	29.7	0.5
Potassium	16.7413	2.8	1.5	4	20.4
Lithium	0.7485	<0.1	<0.1	<0.1	<0.1
Magnesium	2.077491	<0.1	<0.1	<0.1	0.5
Manganese	6.296724	1.3	2.1	33.5	0.4
Molybdenum	<0.1	<0.1	3.6	<0.1	<0.1
Sodium	2.14403	2.3	1.8	1.9	5
Nickel	<0.1	0.2	<0.1	<0.1	6.9
Lead	<0.1	<0.1	<0.1	<0.1	0.3
Silicon	3.111308	18.1	4	0.3	2.3
Titanium	0.943458	0.5	0.6	0.3	6.5
Vanadium	<0.1	<0.1	0.4	<0.1	<0.1
Zinc	0.124657	<0.1	0.3	0.2	0.2
Fluoride ions	19.35594	1.5	0.1	2.9	30.4
Chromium (VI)	N/A	2.3	0.5	0.3	0

### Nasal PAFR protein expression in vivo

Non-smoking welders and non-smoking controls were recruited to the study. Participants were screened using a questionnaire which included information about age, when the participant last carried out welding activities and how many hours a week they are involved in welding activities in a typical week. Participants with a previous doctor diagnosis of asthma, chronic obstructive pulmonary disease, chronic bronchitis or metal fume pneumonia, who had ever had a pneumococcal vaccination, currently using a nasal spray, or had had recent nasal surgery were excluded. Written informed consent to participate was obtained from all participants. The study received ethical approval from the national health service (NHS) Health Research Authority North East—Newcastle & North Tyneside 1 Research Ethics Committee (reference: 15/NE/0237).

Nasal epithelial cell sampling was carried out using a Rhinoprobe (VWR, Lutterworth, UK). An epithelial sample was obtained from both nostrils from each participant. Samples were pooled for assessment of constitutive PAFR expression by flow cytometry (described below).

### Respiratory cell lines and *Streptococcus pneumoniae*

A549 cells, an alveolar epithelial cell line (Sigma Aldrich, Dorset, UK), were maintained in Dulbecco’s Modified Eagle’s Medium (DMEM) containing HEPES (4-(2-hydroxyethyl)-1-piperazineethanesulfonic acid), supplemented with fetal bovine serum (FBS), L-glutamine and antibiotics (Lonza, Basel, Switzerland). Passage number was less than 20. BEAS-2B, a bronchial epithelial cell line, was a gift from Dr Nicolas Mercardo (National Heart and Lung Institute, Imperial College London, UK). BEAS-2B cells were maintained in Roswell Park Memorial Institute-1640 media containing HEPES (RPMI, Life Technologies, Paisley, UK) supplemented with FBS L-glutamine and antibiotics. Passage number was less than 20. Cells were maintained in an incubator set to 37°C and 5% CO_2_. The type 2 *Streptococcus pneumoniae* (*S*. *pneumoniae*) encapsulated strain D39 was purchased from the National Collection of Type Cultures (NCTC 7466, Central Public Health Laboratory, UK) and grown in liquid culture brain heart infusion broth (Oxoid, Basingstoke, UK). Pneumococci were grown at 37°C in a water bath to mid-logarithmic phase (optical density 600 = 0.4 to 0.6) before use in cell infection experiments.

### Welding fumes and pneumococcal growth

Pneumococci were grown for 3h in 200μg/ml WF at 37°C in a water bath. Samples were taken every hour. Serial dilutions of the samples were plated on brain heart infusion agar containing 5% horse blood (Oxoid, Basingstoke, UK) and colony forming unit count/mL (CFU) assessed.

### Pneumococcal infection of respiratory cell lines

Pneumococcal infection of respiratory cells was assessed using our standard method[10]. Briefly, confluent monolayers of cells were cultured with WF (200 μg/mL) for 2h, washed, then infected with *S*. *pneumoniae* at a multiplicity of infection (MOI) of 100. After 2 h, cells were vigorously washed and lysed using sterile distilled water. Serial dilutions of the samples were plated on brain heart infusion agar containing 5% horse blood (Oxoid, Basingstoke, UK) and colony forming unit count/mL (CFU) assessed. In this infection assay, increased CFU counts reflect both increased adhesion of pneumococci to cells and increased penetration of pneumococci into cells [10].

### Flow cytometry

A549 and BEAS-2B cells were incubated with 200 μg/ml MS-WF +/- NAC. For WF exposed cells, cells were exposed to WF for 2h. For NAC treated cells, cells were pre-treated with NAC (5mM) for 30mins in an incubator and then exposed to WF + NAC[17]. The analysis was carried out on a BD FACS Canto II machine using BD FACSDiva software (BD Biosciences, Oxford, UK). Median florescence intensities of the controls and exposed cells for each fluorophore are compared as described below.

### PAFR surface protein expression

Nasal epithelial cells from welders were stained with PAFR primary antibody (1:200, Abcam, Cambridge, UK) for 1h with shaking at room temperature (RT). In order to analyse PAFR expression specifically in epithelial cells, a marker for epithelial cells, E-cadherin (primary antibody used at 1:100, Abcam, Cambridge, UK) was concurrently used. Cells were then washed and stained with secondary antibodies conjugated to Alexa Fluor 488 for PAFR expression (1:3000, Abcam, Cambridge, UK) and conjugated to APC for E-cadherin expression (1:1500, Abcam, Cambridge, UK) for 30min with shaking at RT. Cells were washed and analysed as described before. An isotype control for PAFR was used to exclude any non-specific staining. The resulting median florescence intensity was measured.

Cell lines were stained with PAFR primary antibody (1:200, Abcam, Cambridge, UK) for 1h with shaking at room temperature (RT). Cells were then washed and stained with secondary antibody conjugated to Alexa Fluor 488 (1:3000, Abcam, Cambridge, UK) for 30min with shaking at RT. Cells were washed and analysed as described before. The resulting median florescence intensity was measured.

### HIF-1α protein expression

Cells were fixed in 2% paraformaldehyde (PFA) (Sigma Aldrich, Dorset, UK) for 10min at RT, and permeabilised with 0.5% Tween-20 solution (Sigma Aldrich, Dorset, UK) for 15min at RT. Cells were stained with HIF-1α primary antibody (1:100 in permeabilisation solution, Santa Cruz Biotech, Wembley, UK) for 1h with shaking at RT. Cells were then washed and stained with secondary antibody conjugated to APC (1:1500 in permeabilisation solution, Abcam, UK) for 30min at RT with shaking. Cells were washed and analysed as described before. The resulting median florescence intensity was measured. This methodology was developed and optimised in accordance with the manufacturer’s recommendations (Abcam, Cambridge, UK).

### Transfection

Knockdown/overexpression at the mRNA level was assessed using real time polymerase chain reaction (RT-PCR). Cells were lysed for RNA extraction using the RNAeasy kit (Qiagen, Manchester, UK). cDNA was produced using the SuperScript Vilo MasterMix (Thermo Fisher Scientific, UK) according to manufacturer’s instructions. RT-PCR was performed and analysed using the deltadeltaCt method using the Taqman gene expression mastermix (Thermo Fisher Scientific, Paisley, UK) and Taqman primers and probes for HIF-1α and PAFR (Thermo Fisher Scientific, Paisley, UK), according to manufacturer instructions. β-actin was used as the housekeeping gene. The siRNA knockdown and overexpression methodology, described below, were developed and optimised based on manufacturer recommendations (Thermo Fisher Scientific, Paisley, UK).

### HIF-1α siRNA knockdown

Confluent monolayers of A549 and BEAS-2B cells were transfected with manufacturer validated HIF-1α siRNA (100nM, Thermo Fisher Scientific, Paisley, UK) against HIF-1α using lipofectamine (1μl/well in a 24 well plate, Thermo Fisher Scientific, Paisley, UK) in reduced serum media (4% FBS) for 48h. The siRNA complexes were formed in serum free media according to manufacturer recommendations. After 48h an infection assay was performed as described before. Two different siRNAs were used, to control for off target effects, but not in conjunction with each other. Infection data for both siRNAs were pooled for analysis. A negative control siRNA (Thermo Fisher Scientific, Paisley, UK) was used in all experiments.

### HIF-1α overexpression

50–80% confluent monolayers of A549 and BEAS-2B cells were transfected with HIF-1α plasmid (Sino Biological, Beijing, China) containing a Hygromycin B resistance gene. Transfection was carried out in T25 flasks using 10μg of plasmid and 100μl of lipofectamine in serum free media as per manufacturer’s recommendations. After 3 days the cells were passaged in Hygromycin B (VWR, Lutterworth, UK) containing media to select for cells containing the plasmid (untransfected cells were sensitive to the antibiotic). After 3 days cells were washed to remove the antibiotic and an infection assay performed as described before. Untransfected cells were used as a control.

### Statistical analysis

Statistical analysis was conducted using GraphPad Prism version 6 (GraphPad Software, La Jolla, USA). Data were obtained from at least 3 separate experiments done at different times with each data point representing the mean of at least 3 technical replicates within an experiment, unless otherwise stated. Data are summarised by mean ± SEM, and analysed by either t test, or one-way ANOVA and multiple comparisons tests. For analysis of the effect of WF on pneumococcal growth, a 2-way ANOVA was used. A p value <0.05 was considered significant.

## Results and discussion

### Nasal PAFR expression in welders

**Fig 1:**
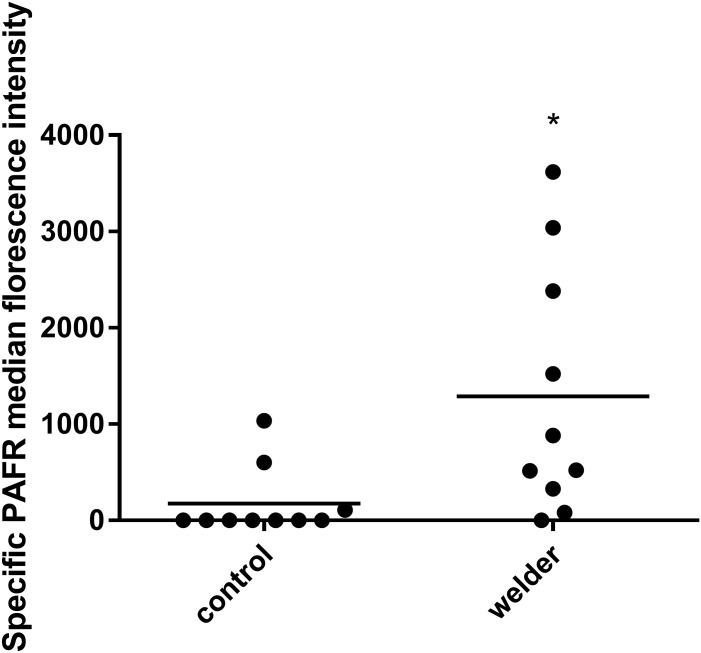
Constitutive expression of PAFR in nasal samples of welders vs controls. Nasal epithelial cells were obtained, stained for PAFR and analysed using flow cytometry. Constitutive expression of PAFR in the nasal epithelium of welders was significantly higher compared to controls (*p<0.05). Data are from 10 welders vs 10 controls and are normalised to isotype control for each sample. Data are compared using t test.

Nasal epithelial cells were obtained from 10 non-smoking welders and 10 non-WF exposed non-smoking controls. All participants were male and between 20 and 64 years of age. In welders, the mean (SEM) duration of welding activities per week was 27.2 ± 3.1 h. All welders had performed welding activities within 4 weeks of nasal sampling. Constitutive PAFR protein expression of controls was low, with PAFR expression above isotypic control expression found in 3/10 controls. By contrast, specific PAFR expression was found in 9/10 welders. PAFR expression was higher in welders compared with controls (*p<0.05, Fig 1).

### WF and infection *in vitro*

**Fig 2:**
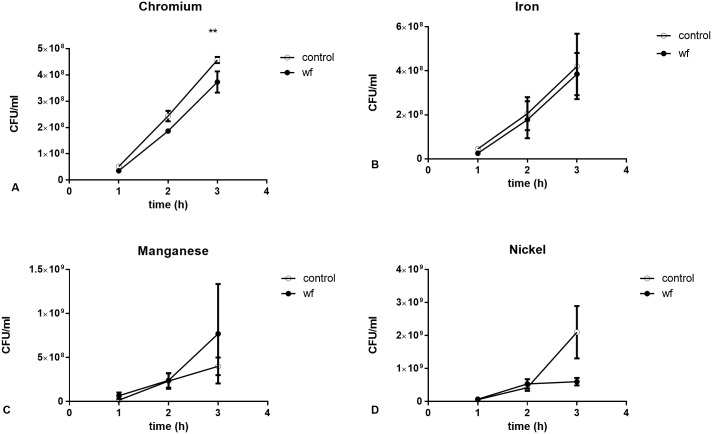
Effect of chromium-, iron-, manganese- and nickel-rich WF samples on pneumococcal growth. Pneumococci were grown for 3h in WF and samples were taken every hour to measure CFU/ml. Pneumococci grown for 3h in A) chromium-rich WF had significantly lower CFU counts compared to controls (**p<0.01). Pneumococci grown for 3h in B) iron-rich WF, C) manganese-rich WF and D) nickel-rich WF did not have significant differences in CFU counts compared to controls. Data are from 3 separate experiments. Each data point represents one sample. Data are matched and compared using 2 way ANOVA.

To assess the effect of WF on pneumococcal growth, bacteria were grown in 200 μg/ml either chromium-rich, iron-rich, manganese-rich, or nickel-rich WF for 3h. Chromium-rich WF significantly reduced bacterial growth (**p<0.01, Fig 2). Iron-, manganese- and nickel-rich WF (Fig 2) did not stimulate bacterial growth over the 3h but also did not inhibit it.

**Fig 3:**
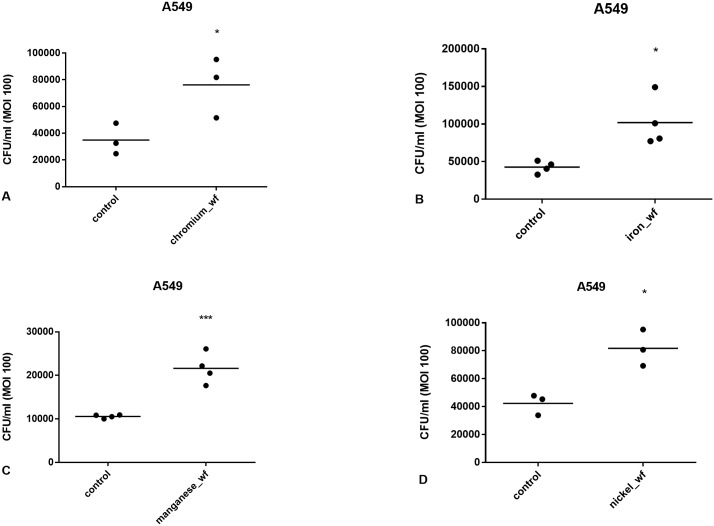
Effect of A) chromium-, B) iron-, C) manganese- and D) nickel-rich WF on pneumococcal infection in A549 cells. Cells were exposed to WF for 2h. The WF was then washed off and the cells infected with pneumococci at MOI 100. Loosely adherent bacteria were washed off and the cells lysed to analyse infection by measuring CFU/ml. All WF samples significantly increased pneumococcal infection of cells (*p<0.05, *p<0.05, ***p<0.001, *p<0.05 respectively). Data are from 3 separate experiments. Each data point represents the mean of 3 technical replicates within an experiment. Data are compared using t test.

**Fig 4:**
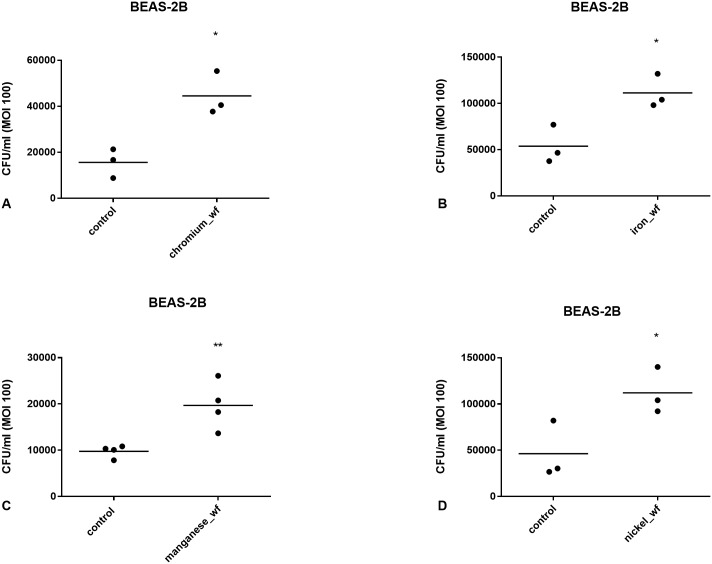
Effect of A) chromium-, B) iron-, C) manganese- and D) nickel-rich WF on pneumococcal infection in BEAS-2B cells. Cells were exposed to WF for 2h. The WF was then washed off and the cells infected with pneumococci at MOI 100. Loosely adherent bacteria were washed off and the cells lysed to analyse infection by measuring CFU/ml. All WF samples significantly increased pneumococcal infection of cells (*p<0.05, *p<0.05, **p<0.01, *p<0.05 respectively). Data are from 3 separate experiments. Each data point represents the mean of 3 technical replicates within an experiment. Data are compared using t test.

To assess the effect of WF composition on pneumococcal infection of respiratory cells *in vitro*, cells were exposed to 200 μg/ml WF for 2h. Chromium-rich, iron-rich, manganese-rich, and nickel-rich WF significantly stimulated bacterial infection in A549 (*p<0.05, *p<0.05, ***p<0.001 and *p<0.05 respectively, Fig 3) and BEAS-2B cells (*p<0.05, *p<0.05, **p<0.01, *p<0.05 respectively, Fig 4).

### MS-WF and HIF-1α protein expression

**Fig 5:**
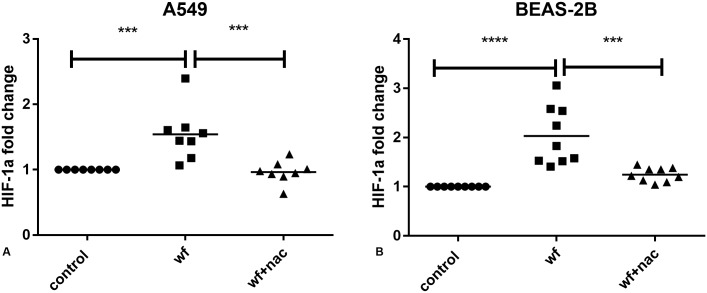
Effect of MS-WF exposure +/- NAC treatment on HIF-1α levels in A) A549 and B) BEAS-2B cells. Cells were exposed to MS-WF +/- NAC as described previously, stained for HIF-1α and analysed by flow cytometry. In A) A549 cells, MS-WF exposure significantly increased HIF-1α levels compared to controls (***p<0.001) which were attenuated in NAC treated cells compared to untreated cells (***p<0.001). In B) BEAS-2B cells, MS-WF exposure significantly increased HIF-1α levels (****p<0.0001) which were attenuated by NAC treatment (***p<0.001). Data are from at least 3 separate experiments. Each data point represents a single experiment. Data are compared using 1 way ANOVA followed by Sidak’s multiple comparison tests.

A549 and BEAS-2B cells were incubated with 200 μg/ml MS-WF (2 h) +/- NAC 30 min pre-exposure and during exposure. MS-WF exposure upregulated HIF-1α protein expression in both A549 (***p<0.001, Fig 5) and BEAS-2B cells (****p<0.0001, Fig 5). MS-WF-stimulated HIF-1α expression in both cell types was attenuated by NAC treatment (***p<0.001, Fig 5).

### MS-WF and PAFR protein expression

**Fig 6:**
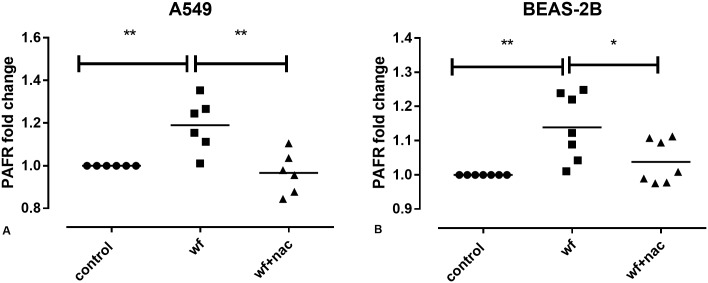
Effect of MS-WF exposure +/- NAC treatment on PAFR levels in A) A549 and B) BEAS-2B cells. Cells were exposed to MS-WF +/- NAC as described previously, stained for PAFR and analysed by flow cytometry. In A) A549 cells, MS-WF exposure significantly increased PAFR levels compared to controls (**p<0.01) which were attenuated in NAC treated cells compared to untreated cells (**p<0.01). In B) BEAS-2B cells, MS-WF exposure significantly increased PAFR levels (**p<0.01) which were attenuated by NAC treatment (*p<0.05). Data are from at least 3 separate experiments. Each data point represents a single experiment. Data are compared using 1 way ANOVA followed by Sidak’s multiple comparison tests.

A549 and BEAS-2B cells were incubated with 200 μg/ml MS-WF 2 h +/- NAC 30 min pre-exposure and during exposure. MS-WF exposure upregulated PAFR protein expression in both A549 (**p<0.01, Fig 6) and BEAS-2B cells (**p<0.01, Fig 6). MS-WF-stimulated PAFR expression was attenuated by NAC treatment (**p<0.01 in A549s; *p<0.05 in BEAS-2Bs, Fig 6).

### Knockdown and overexpression of HIF-1α

HIF-1α knockdown and overexpression was achieved in all experiments as shown in Table 2. The mean reduction with knockdown, assessed by RT-PCR, was 37.7±10.6%, and mean overexpression was 79928 ± 37349%.

**Table 2:** % of HIF-1α knockdown and PAFR mRNA expression in A549 and BEAS-2B cells. HIF-1α knockdown and corresponding and PAFR mRNA % levels are presented in Table 2.

A549 HIF-1α knockdown	A549 PAFR mRNA	BEAS-2B HIF-1α knockdown	BEAS-2B PAFR mRNA
24.09097947	78.49636258	1.516219866	45.10562907
50.73395139	49.35088173	42.39897409	20.66404981
20.67044122	41.88708543	58.9193261	56.80429252
		93.15121584	54.50410123
		10.32677	92.59121153

Compatible with a role of HIF-1α in controlling PAFR expression, HIF-1α knockdown reduced PAFR mRNA expression in unexposed respiratory cells, as shown in Table 2, to 54.9±7.8% compared with samples transfected with negative control siRNA. Conversely, HIF-1α overexpression increased mean PAFR mRNA expression to 542.6 ± 216.4% compared to controls.

HIF-1α overexpression and corresponding and PAFR mRNA % levels are presented in Table 3.

**Table 3:** % of HIF-1α overexpression and PAFR mRNA expression in A549 and BEAS-2B cells.

A549 HIF-1α overexpression	A549 PAFR mRNA	BEAS-2B HIF-1α overexpression	BEAS-2B PAFR mRNA
178141.2457	1042.077658	87474.19431	288.8513347
53924.37783	749.8648897	174.797213	89.76927162

**Fig 7:**
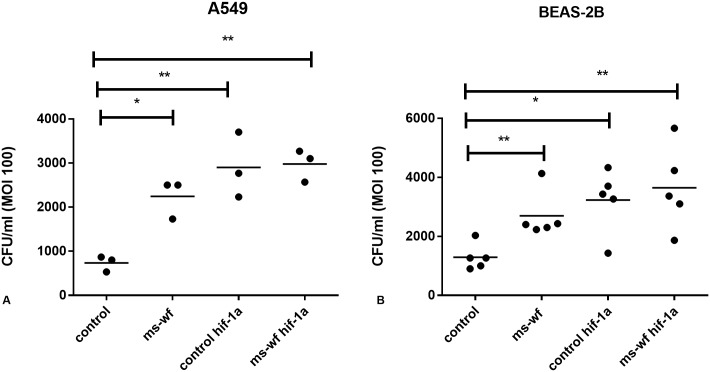
Effect of HIF-1α siRNA knockdown on pneumococcal infection in unexposed and MS-WF exposed A) A549 and B) BEAS-2B cells. Cells were transfected with either HIF-1α or negative control siRNA for 48h. An infection assay as described in the Methods section was then performed to measure CFU/ml. In A) A549 cells, HIF-1α knockdown significantly increased pneumococcal infection in unexposed cells and this was unaffected by MS-WF exposure (**p<0.01, **p<0.01 respectively). In B) BEAS-2B cells, HIF-1α knockdown significantly increased pneumococcal infection in unexposed cells and this was unaffected by MS-WF exposure (*p<0.05. **p<0.01 respectively). MS-WF alone significantly increased pneumococcal infection in A) A549 (*p<0.05) and B) BEAS-2B cells (**p<0.01). HIF-1α knockdown was achieved in all experiments. PAFR expression was, on average, 54% in all HIF-1α knockdown samples compared to controls. Data are from at least 3 separate experiments. Each data point represents the mean of 3 technical replicates within an experiment. Data are compared using 1 way ANOVA followed by Dunnett’s multiple comparison test.

In unexposed respiratory cells, HIF-1α knockdown (which reduced PAFR expression) increased pneumococcal infection compared to cells transfected with negative control siRNA (**p<0.01 in A549s, *p<0.05 in BEAS-2Bs, Fig 7). MS-WF stimulation significantly increased pneumococcal infection of cells transfected with negative control (*p<0.05 in A549s, **p<0.01 in BEAS-2Bs, Fig 7) and HIF-1α siRNA (**p<0.01 in A549s, **p<0.01 in BEAS-2Bs, Fig 7). However, MS-WF did not further stimulate pneumococcal infection in HIF-1α knockdown cells (Fig 7).

**Fig 8:**
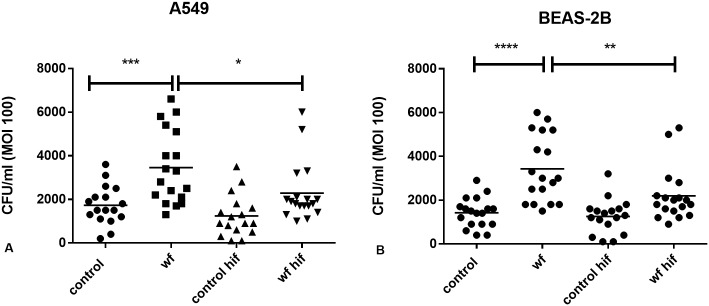
Effect of HIF-1α overexpression on pneumococcal infection in unexposed and MS-WF exposed A) A549 and B) BEAS-2B cells. Cells were transfected with HIF-1α plasmid. Plasmid uptake was selected for using an antibiotic as described in the Methods section. The antibiotic was washed off and an infection assay was performed to measure CFU/ml. HIF-1α overexpression was protective against WF-stimulated infection in A) A549 cells (*p<0.05) and B) BEAS-2B cells (**p<0.01). MS-WF alone significantly increased pneumococcal infection in normal A) A549 (***p<0.001) and B) BEAS-2B cells (****p<0.0001). HIF-1α overexpression was achieved in all experiments. PAFR expression was, on average, 542% in all HIF-1α overexpression samples compared to controls. Each data point represents a single experiment. Data are analysed using 1 way ANOVA followed by Sidak’s multiple comparison test.

Pneumococcal infection in HIF-1α overexpressing, unexposed cells, was similar to pneumococcal infection in cells with unaltered HIF-1α expression. MS-WF stimulation significantly increased pneumococcal infection cells with unaltered HIF-1α expression (***p<0.001 in A549s, ****p<0.0001 in BEAS-2Bs, Fig 8). HIF-1α overexpression was protective against MS-WF stimulated pneumococcal infection (*p<0.05 in A549, **p<0.01 in BEAS-2Bs, Fig 8).

In this study we found that nasal PAFR expression is increased in welders, that a range of WF with different metal compositions stimulate pneumococcal adhesion to respiratory epithelial cells *in vitro*, and that WF, via oxidative stress induction of HIF-1α expression, regulates PAFR-dependent pneumococcal infection. We also found evidence for a paradoxical role of HIF-1α in mediating PAFR-independent basal pneumococcal infection in unexposed respiratory cells.

Adherence of pneumococci to lower respiratory cells is a first step in the development of airway infection leading to pneumonia[18]. For *S*.*pneumoniae* (and other phosphorylcholine expressing bacteria, such as nontypeable *Haemophilus influenzae* and Acinetobacter species), infection of lower respiratory cells is facilitated by an interaction between bacterial phosphorylcholine and PAFR expressed on host cells[9,19,20]. We have previously reported increased lower respiratory cell PAFR expression in active smokers[12]. This study extends this observation to an occupational group that is also at increased risk of pneumococcal pneumonia. Although it is unclear in the present study whether increased nasal epithelial PAFR expression is associated with increased lower respiratory cell PAFR, these data are compatible with strong levels of bronchial PAFR immunoreactivity (compared with a non-exposed individual) in a lung biopsy specimen from a single welder[10]. Furthermore, since welders were sampled at least 24 h post, and within a month of WF exposure upregulation of nasal PAFR in welders suggests a persistent effect, an observation compatible with epidemiological data suggesting that increased pneumococcal infection risk in welders diminishes one year post cessation of WF exposure [5]. Since respiratory epithelial PAFR expression is upregulated by smoking[12], a limitation of the present study is that although we recruited non-smoking participants we did not objectively rule out smoking e.g. by urinary cotinine or breath carbon monoxide.

Our previous study focused on MS-WF[10], but in this study we found that pneumococcal infection was stimulated by a wide range of WF with differing proportions of metals most commonly found in WF samples in high abundance[21–24]. Furthermore, by incubating WF with pneumococci, we excluded a confounding effect of WF stimulation of pneumococcal growth *per se* (which could result in higher CFU in the bacterial infection assays). Specifically, we found no evidence for the hypothesis that WF iron promotes pneumococcal growth[2].

It is well established that HIF-1α plays a critical role in the cellular response to oxidative stress[25]. Additionally, we have previously shown that MS-WF had increased oxidative potential (OP) compared with low-OP carbon black and high-OP urban particulate matter[10]. Our *in vitro* results support the hypothesis that a pathway for WF-induced susceptibility to pneumococcal infection is that exposure to WF, via induction of oxidative stress in respiratory cells, increases HIF-1α expression, and that this in turn upregulates PAFR-dependent pneumococcal infection. Specifically we found that WF stimulates both HIF-1α and PAFR expression in respiratory cell lines, and that the WF-stimulated increase in these proteins is attenuated by the anti-oxidant NAC. The capacity of HIF-1α to directly control PAFR expression was confirmed by HIF-1α knock down experiments, which showed that HIF-1α knock down reduced PAFR mRNA expression. Conversely, HIF-1α overexpression increased PAFR mRNA expression. Indeed these data are compatible with the direct link between HIF-1α and PAFR that is reported in both intestinal and cancer cells[14,26].

We unexpectedly found that in respiratory cells that were not exposed to WF, changes in HIF-1α have a paradoxical effect on pneumococcal infection, i.e. although PAFR was reduced in HIF-1α knockdown cells and PAFR expression was increased in cells overexpressing HIF-1α, pneumococcal infection did not move in parallel with PAFR. Transfection itself did not alter the response of cells since increased PAFR-dependent pneumococcal infection in response to WF was found in cells transfected with control siRNA. In the first publication to show that PAFR mediates pneumococcal infection in respiratory cells, Cundell et al[9] found that PAFR is not a major receptor for “basal” pneumococcal adhesion and infection in unstimulated cells. Indeed, we previously found that blocking PAFR does not attenuate “basal” pneumococcal adhesion and infection in unstimulated respiratory epithelial cells, [10]. Our unexpected results suggest that, although HIF-1α is a major signaling pathway for PAFR-dependent adhesion in WF-exposed cells, it suppresses “basal” adhesion and infection via an as yet unknown, PAFR-independent, mechanism. Indeed, using human uroepithelial cells and a mouse model of urinary tract infection it has previously been shown HIF-1α accumulation was protective against uropathogenic *E*. *coli* infection, whereas increased amounts of bacteria were recovered from bladders of mice with HIF-1α deletion[27]. Additionally, basal HIF-1α expression maintains intestinal human β defensin-1 and murine defensin expression, which are key anti-microbial peptides[28].

## Conclusions

We found increased expression of PAFR, a receptor for pneumococcal infection, in nasal epithelial cells from welders. We therefore speculate that nasal PAFR expression is a biomarker for increased susceptibility to pneumococcal infection in welders and may identify those requiring pneumococcal vaccination. Exposing respiratory cells to WF *in vitro*, we found a major role for HIF-1α in mediating oxidative-induced PAFR expression. Indeed, increased HIF-1α expression has been found in other exposure settings such as cigarette smoke extract exposure of lung cells and zinc oxide nanoparticle exposure of kidney cells, which indicates that it may be a common exposure response mechanism[29,30]. We also unexpectedly found that HIF-1α mediates PAFR-independent pneumococcal infection in unstimulated respiratory cells.
